# A non-AI preliminary algorithm for the prediction and detection of highly pathogenic African swine fever in pigs using health monitoring collars

**DOI:** 10.1017/awf.2026.10060

**Published:** 2026-01-28

**Authors:** Rachel Layton, David Beggs, Peter Mansell, Andrew Fisher, Daniel Layton, Brint Gardner, David Williams, Kelly Stanger

**Affiliations:** 1 Australian Animal Health Laboratory, CSIRO Australian Centre for Disease Preparedness Business Unit, Geelong, VIC, Australia; 2 Faculty of Science, Melbourne Veterinary School, The University of Melbourne, Werribee, VIC, Australia; 3 Health and Biosecurity, CSIRO Australian Centre for Disease Preparedness Business Unit, Geelong, VIC, Australia; 4 Information Management and Technology, CSIRO, Clayton, VIC, Australia

**Keywords:** Animal welfare, biomarkers, HRV, PetPace^TM^, pulse, respiratory rate

## Abstract

Collar monitoring devices are used in animals for the minimally invasive collection of physiological data, using software and algorithms to provide general health trends. There is potential to utilise the raw data collected from these devices to improve animal monitoring strategies and intervention points in animal disease studies. We aimed to develop an algorithm for the early detection of highly pathogenic African swine fever disease in research pigs (*Sus scrofa*), using data collected via modified PetPace^TM^ health monitoring collars. Pigs from two other studies (n = 6 per study, total n = 12) were opportunistically available and fitted with collar monitors for the daily collection of pulse rate, respiratory rate and heart rate variability, prior to and after experimental challenge with highly pathogenic African swine fever virus. Collar monitors detected a decreased mean, and increased variability, of pulse rate and heart rate variability in pigs post-challenge, which was not detected by single daily point-in-time measurements. The incidence of abnormal pulse rate, respiratory rate and heart rate variability readings increased in pigs after infection with highly pathogenic African swine fever, with increasing abnormal readings occurring both prior to the onset of, and during, clinical disease. A preliminary non-AI algorithm utilising these data detected disease in 100%, and predicted disease onset in 67%, of infected pigs. This paper describes how health-monitoring collars can be used to improve the early detection of African swine fever disease in pigs. Additionally, it provides a potential framework for developing and using non-AI algorithms in other disease models, to enhance animal monitoring and welfare outcomes in research animals.

## Introduction

Health-monitoring devices are increasingly utilised by farmers, veterinarians, researchers and pet owners to monitor animal health. These devices commonly take the form of implantable or wearable monitors that regularly and automatically record activity, behaviour, or clinical parameters, such as pulse rate, heart rate variability and respiratory rate (Neethirajan 2017). The emergence and progression of precision livestock farming for the early detection of on-farm health issues has led to substantial developments in real-time monitoring, using biosensors and algorithms (Vaintrub *et al.* 2021; Tzanidakis *et al.* 2023). Similar devices have also been developed for use by pet owners and veterinarians to monitor the health and welfare of companion animals, most commonly in the form of collar monitors (Rowlison *et al.* 2022; Schork *et al.* 2023). While the uptake of health-monitoring technologies for the collection of data in research animals has not been as rapid, there are increasing opportunities to utilise these devices to improve welfare and scientific outputs in laboratory animals (Layton *et al.* 2023).

Infectious disease research requires careful and intensive animal monitoring, with some diseases under investigation progressing rapidly to severe morbidity and mortality. This can make balancing the welfare of animals with scientific objectives challenging (Richter *et al.* 2024). There is an opportunity to utilise health-monitoring devices to measure physiological parameters in animal models of infectious disease, to improve monitoring and reduce the animal welfare impacts of high-impact diseases. The raw data collected by these devices have the potential to be used for developing algorithms for disease detection and prediction, which are often developed using AI and machine learning-based methods (Mei *et al.* 2019; Carslake *et al.* 2020). However, the use of AI and machine learning typically requires large datasets for algorithm development and training, the collection of which is not always feasible. The development and continued training of AI-based algorithms also require technical expertise that is not always readily available (Sarker 2021). This can limit the use of algorithms as a tool for animal monitoring in many fields of research.

African swine fever (ASF) is an infectious, haemorrhagic, viral disease affecting members of the family Suidae. ASF outbreaks can cause up to 100% mortality rate, and the economic and welfare consequences make it a commonly studied disease to investigate pathogenesis, infection dynamics and vaccine development (Faverjon *et al.* 2021). The severe clinical presentation and rapid progression of highly pathogenic forms of ASF disease in pigs means that intensive monitoring is critical to protecting laboratory pig welfare (Li *et al.* 2022). In order to continually improve upon monitoring strategies, novel techniques and technologies should be investigated to enhance the prediction and detection of disease onset, progression, and impending humane endpoint.

In this study, we hypothesised that changes in pulse rate, heart rate variability and respiratory rate could be used to detect and predict disease in pigs infected with highly pathogenic ASF virus (ASFV). Pigs that were enrolled in two African swine fever studies for the evaluation of disease dynamics were utilised opportunistically to also investigate collar monitors for physiological data collection and algorithm development. This additional objective was included in these studies as a means of identifying a potential welfare refinement by enhancing methods of animal monitoring, without using additional animals. Modified PetPace™ health-monitoring collars were placed upon and removed from pigs daily for the regular, automatic monitoring of clinical parameters. Data were collected both prior to and after challenge in two pig studies of highly pathogenic ASFV, until humane endpoint was reached. Using these data, we aimed to develop a preliminary non-AI algorithm for the prediction and detection of ASF disease, to improve the monitoring and management of pigs infected with highly pathogenic ASFV. The ability to detect ASF disease using the larger datasets collected via monitors was also compared to a more typical, daily point-in-time monitoring strategy.

## Materials and methods

### Ethics statement

Animal studies were approved by the ACDP (Australian Centre for Disease Preparedness) Animal Ethics Committee (permit numbers 22008 and 2054). Studies were approved to experimentally assess the onset of clinical signs and disease course in pigs. The use of collar monitors for data collection in these studies was approved as an additional opportunistic objective to investigate refinements to pig monitoring in ASF disease studies without increasing animal numbers. All procedures were conducted in accordance with the guidelines of the National Health and Medical Research Council as described in the Australian Code for the Care and Use of Animals for Scientific Purposes (2013).

### Study design

Two studies were conducted to experimentally assess the onset of clinical signs and disease course in pigs challenged with highly pathogenic ASFV (African swine fever virus) Georgia 07’ isolate (Rowlands *et al.* 2008). An additional opportunistic objective was incorporated to use collar monitors to investigate changes in pulse rate, heart rate variability and respiratory rate over the course of infection. Pigs were challenged with a high dose of virus (10⁶ 50% Tissue Culture Infectious Dose [TCID_50_]) in study 1, and a medium dose of virus (10^4^ 50% Tissue Culture Infectious Dose [TCID_50_]) in study 2. For the present study, only the data from pigs that became infected via experimental inoculation on day 0 were included in data analysis — all six pigs in the high dose study, and four pigs in the medium dose study. The remaining two pigs in the medium dose study became infected via secondary contact transmission; these pigs were utilised for algorithm testing.

In both studies, six week old Landrace-cross female pigs (n = 6 per study, total n = 12) were sourced from a local commercial piggery (Geelong, VIC, Australia). Pigs were held under biosafety level 3 containment within the ACDP microbiologically secure animal facility and fitted on arrival with modified PetPace^TM^ health-monitoring collars for the regular, automated recording of physiological parameters. In both studies, pigs underwent acclimation for 14 days, followed by oronasal inoculation on day 0 with the relevant dose of highly pathogenic ASFV. From day 1 until reaching humane endpoint, all pigs were assessed between one to three times daily. Oral swabs, ethylenediaminetetraacetic (EDTA) blood and serum blood were collected once prior to challenge, every two days post-challenge and at humane endpoint for PCR analysis of viral load. Data from the collars were collected between 0800–1600h daily on days –14, –10, –3, –2, –1 and 0, and daily between day 1 and reaching humane endpoint (between days 6 and 10 for pigs included in the data analysis for the present study). Upon reaching humane endpoint, all pigs were humanely killed under general anaesthesia (tiletamine/zolazepam 4.4 mg kg^–1^ [Virbac, Carros, France] and xylazine 2.22 mg kg^–1^ [Troy Animal Health, Glendenning, NSW, Australia]) via IV pentobarbitone 150 mg kg^–1^ (Virbac, Carros, France).

### Animal management and acclimation

Pigs (n = 6 per study) were group-housed in a pen measuring 7.00 × 2.96 m (length × width). The pen was furnished with a rubber mat and a plastic bed containing straw, in addition to enrichment items that were rotated daily (plastic balls, chains and rubber hosing). Barastoc™ pig grower pellets (Ridley Corporation, Melbourne, VIC, Australia) were available *ad libitum* and pen cleaning was conducted daily. Room temperature was maintained at 22°C with an 8-h light:16-h dark cycle. Pigs were housed for 21–25 days, including 14 days prior to ASFV inoculation and 6–10 days after ASFV inoculation.

### PetPace^TM^ health monitoring collars

PetPace^TM^ health monitoring collars (PetPace^TM^ LLC, Burlington, MA, USA) were modified for safe use in pigs as described by Layton *et al.* (2025). Collars were set to log at 5-min intervals for the monitoring of pulse rate, respiratory rate and heart rate variability (recorded as VVTI), and were fitted on pigs between the hours of 0800–1600h daily when staff were present to monitor collar use. Collars were removed daily prior to pigs being left unsupervised to prevent pigs from damaging the collars of their pen-mates. If pigs attempted to chew on the collars of co-housed pigs when wearing the collars supervised, a staff member would walk past the pen to distract the pig until the chewing attempts ceased and pigs lay down to rest.

### Collection of multiple and single daily readings

To assess differences in sensitivity between traditional point-in-time (PIT) measurements and the multiple daily readings collected using the collars, a single PIT daily reading at 1000h was retrospectively identified. This PIT reading was collected via collar monitors for each variable on each day of collar measurements (pulse rate, HRV and respiratory rate). The time of 1000h was selected as it was deemed the most likely time manual measurement of clinical parameters would have occurred, had regular, automated monitoring devices not been utilised. If a reading was not collected from the collar monitors at 1000h, the closest reading to 1000h was selected (± 30 min). This time-point was selected prior to commencing PIT data analysis.

### Virus utilised

Animals in this study were challenged with the highly virulent African swine fever Georgia 2007/1 (GRG/07) isolate (10% spleen homogenate) that has been described previously (Rowlands *et al.* 2008). All *in vitro* and *in vivo* work involving live ASFV was conducted within biosafety level 3 facilities at the ACDP.

### Experimental infection of pigs

Prior to experimental challenge, baseline serum and EDTA whole blood were collected from each pig to confirm that animals were negative for antibody to ASFV and viral DNA using the INGEZIM PPA Compac R.11.PPA.K3 blocking ELISA (Ingenasa, Madrid, Spain), and an ASFV-specific PCR (Thermo Fisher Scientific, Australia). Pigs were also screened for influenza A virus and porcine circovirus types 1 and 2 using in-house PCR and serology tests. On the day of viral challenge, under general anaesthesia, a dose of 10^6^ TCID_50_ (high viral dose) or 10^4^ TCID_50_ (medium viral dose) in 5.0 mL PBS was administered via the oro-nasal route to simulate natural infection. The inoculum was administered to pigs held in dorsal recumbency by slow dropwise instillation of 1.5 mL into each nostril and 2.0 mL into the oral cavity. Back-titrations were performed to confirm the dose. Following inoculation, virus infection was confirmed in all pigs in both high and medium dose groups by qPCR testing of oral swab and whole blood samples.

### Biological sample collection

Samples were collected from pigs under general anaesthetic to investigate viral and immune responses, as part of the broader immunity and pathogenesis study objectives. Samples were collected from all pigs, once prior to viral challenge (day –6), every two days post-challenge, and upon reaching humane endpoint. All samples were collected on sample days between 0900–1100h. On sample collection days, anaesthetic (tiletamine/zolazepam 4.4 mg kg^–1^ and xylazine 2.22 mg kg^–1^) was administered into the hind leg (biceps femoris) via IM injection, using a 19-g 1½’ needle. At each sample collection event a sterile cotton oral swab (Pacific Laboratories, Blackburn, VIC, Australia) was wiped over the tongue and hard palate then placed into a 1-ml collection tube containing viral transport media. Additionally, blood was collected from the cranial vena cava of pigs placed in dorsal recumbency into whole EDTA and serum vacutainer tubes (BD, Franklin Lakes, New Jersey, USA) using a 20-g 1½’ needle. Oral swabs and EDTA blood were kept at 4°C and serum blood at room temperature until processing. Volume of blood collected did not exceed 7.5% of total blood volume per week.

### qPCR analysis

Whole EDTA blood, rope chew oral fluid and oral swabs were processed for real-time PCR (qPCR) by adding 120 μl of each sample to 315 μl of MagMax Lysis/Binding solution (Thermo Fisher Scientific, Australia). Next, 180 μl of lysate was used to extract total nucleic acid using the MagMAX Pathogen RNA/DNA kit (Thermo Fisher Scientific, Australia) in a KingFisher™ Purification System (Thermo Fisher Scientific, Australia), following the manufacturer’s instructions. qPCR was performed using the assay described by Zsak *et al.* (2005) using 5 μL of DNA template, sense and antisense primers (300 nM) and TaqMan® probe (250 nM), and AgPath-ID one-step RT-PCR reagents (ThermoFisher) in a 15 μL reaction volume. Thermocycling conditions were 45°C 10 min, 95°C 10 min, and 45 cycles of 95°C 15 s, 60°C 45 s.

### Disease monitoring and humane endpoints

Disease monitoring occurred once daily until the onset of any clinical disease sign in any pig, after which monitoring was increased to three times daily. Rectal temperatures were taken at each monitoring point. Disease progression and determination of humane endpoints were assessed using the scoring matrix as described in Table 1.

**Table 1.** African swine fever monitoring and humane endpoint assessment in study of pigs using health-monitoring collars

	Mild (score = 1)	Moderate (score = 2)	Severe (score = 3)
Body temperature	≥ 40.0°C in a single day	≥ 40.0°C observed once on each of 2 consecutive days	≥41°C
Body condition	BCS ≥ 2.5 but < 3 Vertebrae, ribs and pelvis palpable	BCS ≥ 2 but < 2.5 Vertebrae, ribs and pelvis are visible; noticeable visual loss of condition	BCS < 2 Vertebrae, ribs and pelvis are easily visualised/ prominent; marked visual loss of condition
Activity	Decreased activity, rouses when approached.	Requires more than one attempt to rouse. Returns quickly to lying down (< 3 min).	Does not rise or ambulate.
Mentation	Slightly delayed response to stimulus	Decreased response to stimuli.	Markedly decreased or no response to stimulation.
Ocular	Mild reddening or swelling of conjunctiva/eyes, excessive lacrimation or other discharge.	Conjunctivitis with scleral haemorrhages.	Conjunctivitis with marked scleral haemorrhages bilaterally and signs of ocular pain (photophobia, blepharospasm, excessive discharge)
Skin and mucous membranes	Mild reddening of extremities or skin patches.	Dark reddening of extremities or skin patches. Mucous membranes (oral, vulva) haemorrhages.	Dark reddening of extremities or skin patches with cyanotic (purple/blue) areas and/or dark/black crusts (scabs)
Digestive	Evidence of diarrhoea on hindlimbs for < 24 h	Faeces on rear with markedly liquid/loose consistency. Occasional vomiting (more than 1).	Loose or liquid faeces on rear with traces of blood seen in faeces or urine, black tarry colour or extensive mucous. Frequent/persistent vomiting.
Respiratory	Mild dyspnoea, slightly increased respiratory rate from normal, or effort noted. Mild serous (clear) discharge	Moderate dyspnoea, moderate effort increased from normal, coughing, abdominal breathing. Evidence of upper respiratory discharge (mucus), traces of blood.	Marked dyspnoea, characterised by increased rate and/or effort from normal, postural adjustments during respiration, severe/persistent coughing. Marked upper respiratory discharges including froth or blood
A cumulative score of 11 will trigger humane endpoint. Pigs may also show a rapid decline caused by ASF. In such cases, animals will be humanely killed. The trigger for humane killing will apply to pigs that progress from a clinical score of ≤4 at the morning check followed by a score of ≥9 at any subsequent check that same day. Humane endpoints: Any score of 11 or above At a PM check, any score of 9 or above IF they scored 4 or less at the AM check (rapid progression) Any of the IMMEDIATE HUMANE KILLING OVERRIDES (listed in the table below) Any of the below signs warrant IMMEDIATE HUMANE KILLING Moribund (incapacitating prostration). Activity OR mentation score = 3 Respiratory failure e.g. gasping for air, cyanotic mucous membranes, paddling etc. Respiratory score = 3 Profuse (copious or heavy bleeding)/rapid haemorrhage Sudden drop > 2°C from fever at previous check Neurological signs, e.g. Incoordination, muscular tremors, paresis, paralysis, convulsions.			

### Definitions of abnormal clinical variables

Reference ranges for normal pulse and respiratory rates of growing pigs were used as described by Abdisa (2017) and Sipos *et al.* (2013). A further reduction of 10% for lower pulse and respiratory rate limits were applied to account for lower values expected to occur when pigs were resting or sleeping (Abdisa 2017). The lower normal limit for heart rate variability of growing, healthy laboratory pigs, measured as vasovagal tonus index (VVTI), was used as described by Layton *et al.* (2025).

### Data inclusion and exclusion for analysis

**Figure 1.**
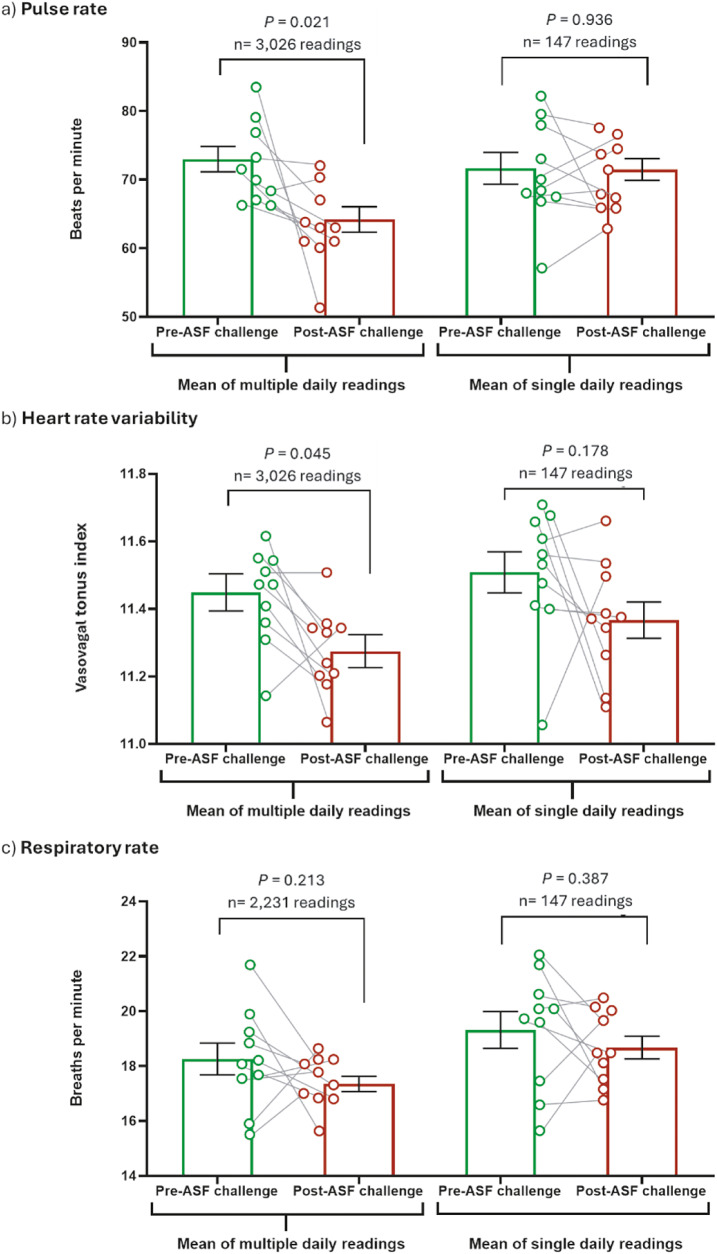
Mean (a) daily pulse rate, (b) heart rate variability (measured as vasovagal tonus index; VVTI) and (c) respiratory rate categorised as pre-ASF challenge days (–14, –3, –2, –1 and 0) and post-ASF challenge days (1–9). Multiple daily readings were collected via modified PetPace^TM^ collar monitors between 0800–1600h. Single daily readings were taken as the daily PetPace^TM^ collar reading closest to 1000h. Data-points represent individual pigs with pre- and post-challenge periods connected by a grey line. Bars represent the mean and error bars represent the standard error of the mean. Pre- and post-ASF challenge means compared using paired parametric *t*-test, with significance set at *P* < 0.05.

**Figure 2.**
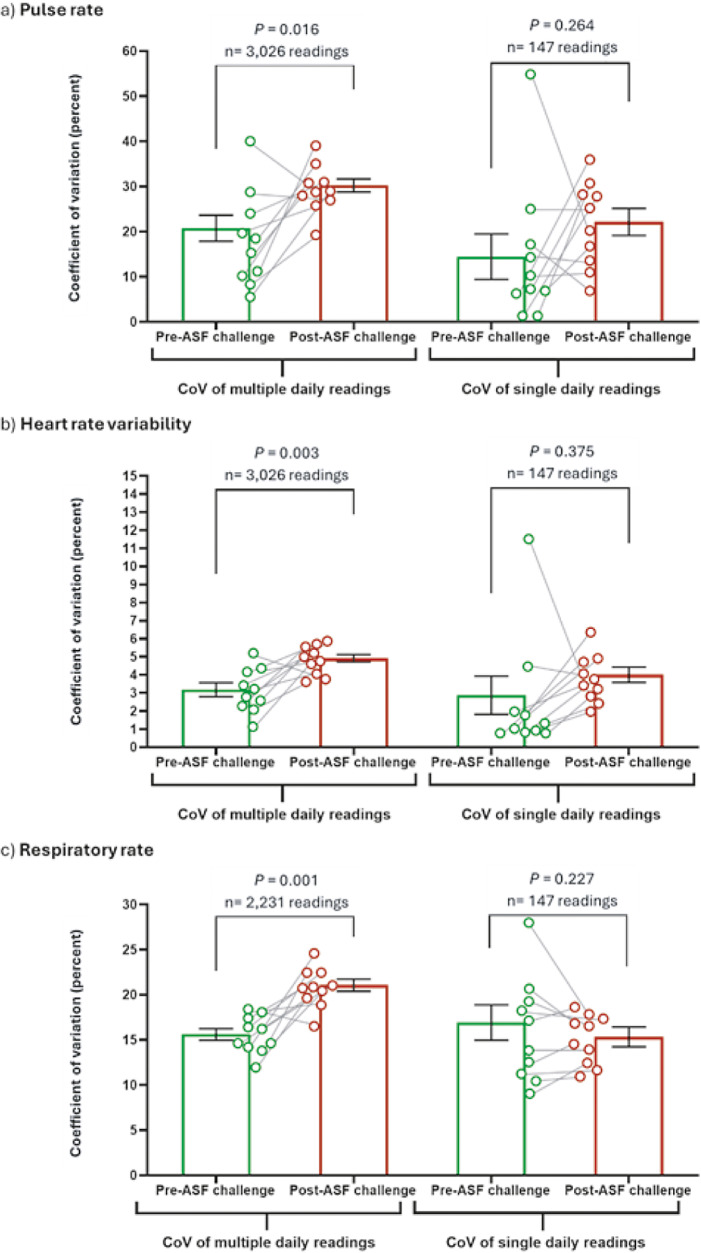
Coefficient of variation (CoV) of (a) daily pulse rate, (b) heart rate variability (measured as vasovagal tonus index; VVTI) and (c) respiratory rate was categorised as pre-ASF challenge days (–14, –3, –2, –1 and 0) and post-ASF challenge (days 1–9). Multiple daily readings were collected via modified PetPace^TM^ collar monitors between 0800–1600h. Single daily readings were taken as the daily PetPace^TM^ collar reading nearest to 1000h. Data-points represent individual pigs, with pre- and post-challenge periods connected by a grey line. Bars represent the mean and error bars represent the standard error of the mean. Pre- and post-ASF challenge CoV compared using paired parametric *t*-test, with significance set at *P* < 0.05.

**Figure 3.**
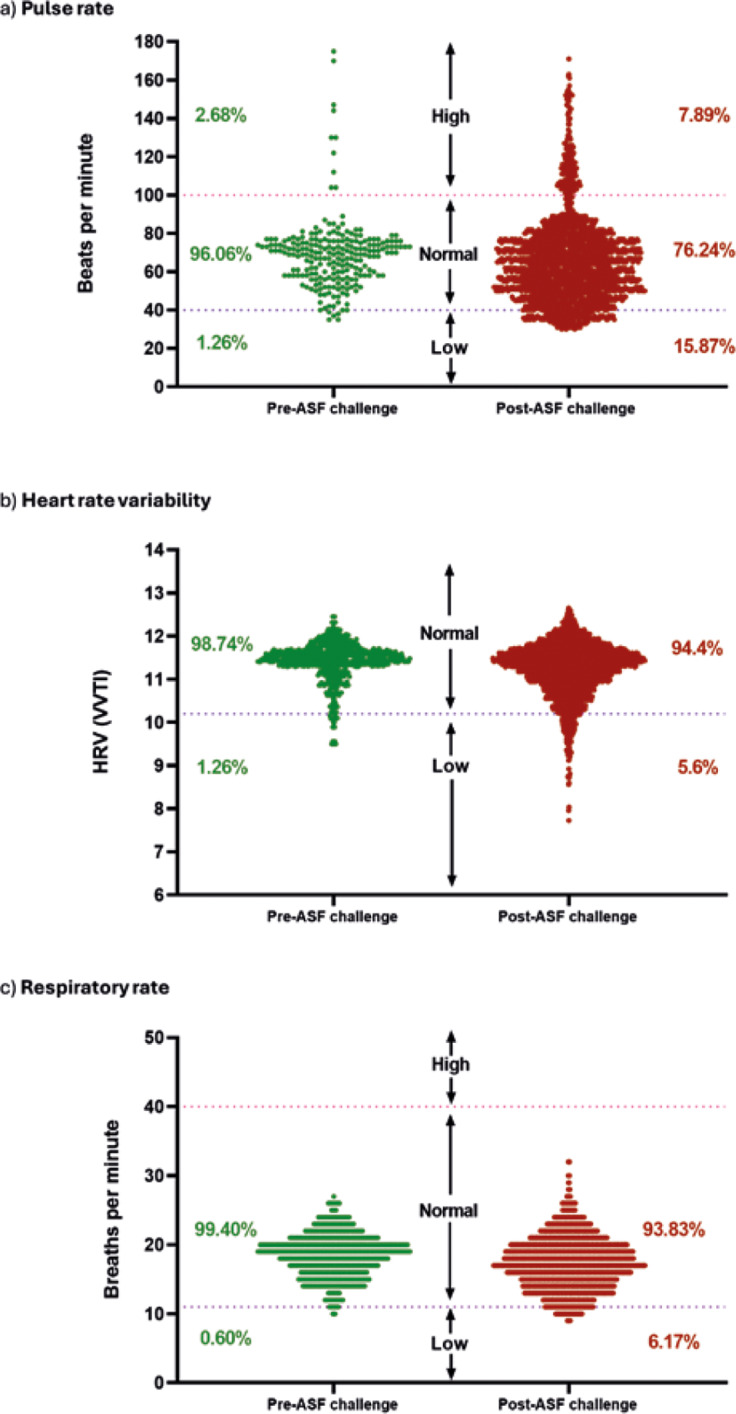
Incidence of (a) abnormal pulse rate, (b) heartrate variability (HRV), measured as vasovagal tonus index (VVTI) and (c) respiratory rate increases in pigs after infection with African swine fever (ASF). Data collected via PetPace^TM^ collar monitors with pre-ASF challenge data collected on days –14, –4, –3, –2, –1 and 0 for all pigs. Post-ASF challenge data collected from day 1 until humane endpoint (days 6–9). Dotted horizontal lines indicate lower (purple) and upper (pink) normal range limits. Respiratory rate data (c) appears as lines due to a reduced number of readings collected compared to pulse rate and HRV.

**Figure 4.**
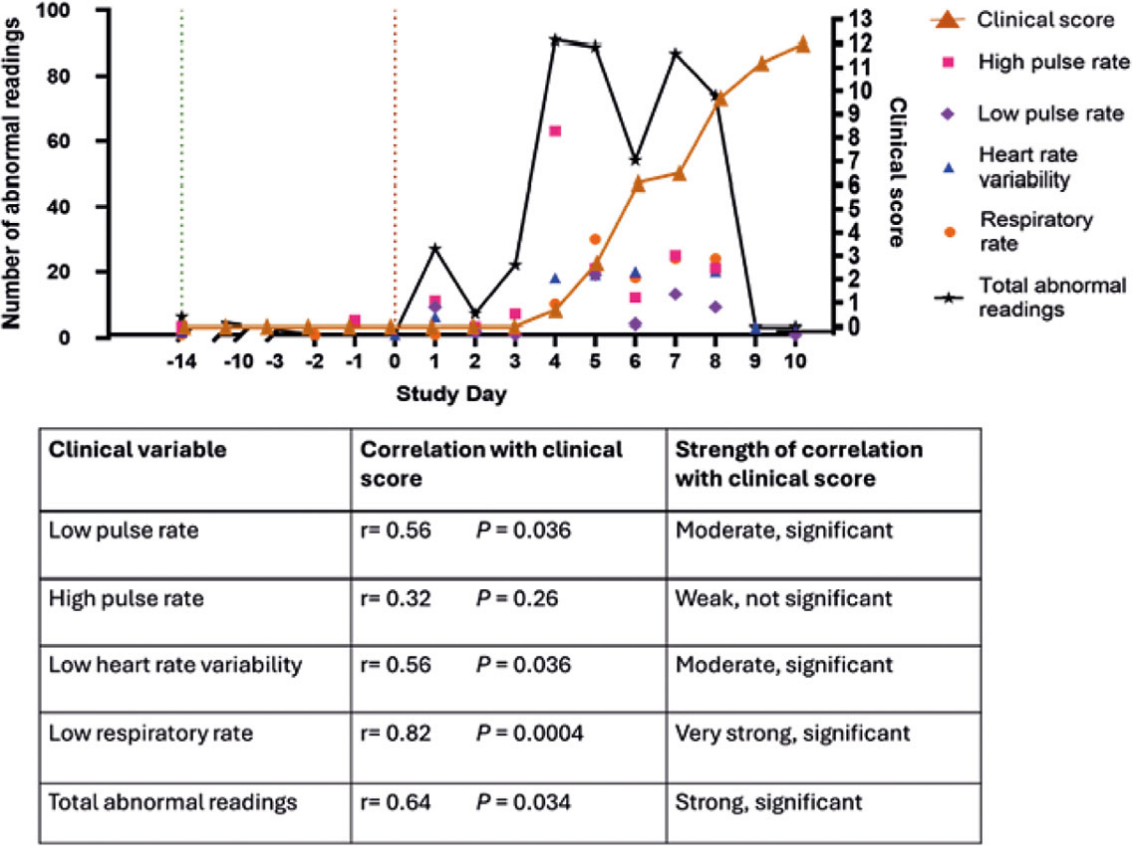
Shows abnormal pulse rate, heart rate variability and increases in respiratory rate prior to and during clinical disease onset in pigs infected with highly pathogenic African swine fever (ASF). Data collection occurred via modified PetPace^TM^ collar monitors between 0800–1600h. Clinical score calculated as the average highest daily score of pigs (n = 9). Green vertical dotted line represents the day of transport and arrival and the red vertical dotted line represents the day of ASF challenge. Correlations between abnormal readings and clinical score calculated using Pearson’s correlation analysis. Significance set at *P* < 0.05.

A total of 12 pigs were inoculated with highly pathogenic ASFV (n = 6 high dose, n = 6 medium dose). Of these 12 pigs, only ten became infected via primary inoculation on day 0 (all six high dose pigs, four of six medium dose pigs). Therefore, only data from these ten pigs were utilised for data analysis in the present study (Figures 1–4, Supplementary Figures 1–6). In addition, one pig in the high viral dose study was euthanased prior to reaching humane endpoint (exhibiting moderate signs of disease). This occurred to ensure it was not left without a companion. Therefore, data from this pig were included in the comparison of means, coefficients of variation and abnormal readings pre- and post-challenge with ASFV (Figures 1–3) but were excluded from all analyses related to daily disease progression to humane endpoint (Figure 4, Supplementary Figures 1–6). All pulse rate, heart rate variability and respiratory rate data were collected between 0800–1600h from pigs in all active states (active, resting and sleeping). Data collected when pigs were anaesthetised for sample collection, and for 3 h after recovery to standing, were excluded from all analyses. This was done to prevent bias associated with the cardiorespiratory effects of general anaesthesia.

### Data inclusion and exclusion for algorithm development and testing

Data from nine of the 12 pigs were utilised to develop a preliminary algorithm for the prediction and detection of ASF clinical disease. These pigs were selected as they all became infected via inoculation at day 0, and reached humane endpoint (five high viral dose, four medium viral dose). Data inclusion and exclusion for algorithm development occurred as described in the previous section. Data from the pigs excluded from algorithm development were instead used to test the algorithm. In addition, PetPace^TM^ collar data were utilised from a previous study of five healthy, uninfected pigs to further test the algorithm. Data from each day pre-challenge in the two ASF studies described (n = 72 days/equivalent to six days of readings from 12 pigs) and from the five healthy pigs in a previous study (n = 99 days/equivalent to 9 or 10 days of readings from five pigs), were used to test for false positive ASF detections. This was a total of 171 days of readings, consisting of 16 days of readings from 17 pigs. A pass was awarded when no ASF disease was predicted or detected by the algorithm. For the prediction of ASF disease, a pass was awarded if the algorithm detected ASF on any day after viral challenge but prior to clinical signs of disease (pass or fail for n = 12 pigs). For the detection of ASF disease, a pass was awarded if the algorithm detected ASF on any day after viral challenge (pass or fail for the same n = 12 pigs). All algorithm development and testing were conducted without the use of artificial intelligence (AI, including machine learning), by retrospectively analysing and observing trends in data prior to challenge and throughout the course of infection. Observations of abnormalities in pulse rate, heart rate variability and respiratory rate were then assessed visually by an operator to identify the combination of metrics that occurred only after pigs had been challenged with ASFV, both prior to and during ASF clinical disease. This was achieved by studying the pulse rate, respiratory rate and HRV data collected in pigs prior to ASF challenge, to determine the normal range for each metric. Data-points that fell outside of these normal ranges were then noted on each day for each pig, both prior to and after ASF challenge. The various combinations of abnormalities per day were then assessed to determine which combinations of abnormal readings occurred only in each of the ASFV-infected pigs. The algorithm was then tested by applying the selected daily combinations of abnormal pulse rate, respiratory rate and HRV readings to pigs (three ASFV-challenged and five healthy) not utilised for algorithm development, to confirm that the algorithm only detected ASFV-infected pigs approaching or at humane endpoint.

### Statistical analysis

All data exploration and statistical analysis were performed using GraphPad Prism® (version 9.1.2, La Jolla, CA, USA). All means, coefficients of variation, standard deviations and percentage of abnormal readings were tested for normality prior to statistical analysis. Normality was confirmed via the D’Agostino and Pearson test and the Shapiro-Wilk test (alpha = 0.05), with all measures passing the normality test and *P*-values not significant (*P* > 0.05). The total number of readings (‘multiple daily readings’) analysed to determine pre- and post-challenge means (Figure 1) and coefficients of variation (Figure 2) were 3,026 each for pulse rate and heart rate variability, and 2,231 for respiratory rate. The number of single daily point-in-time readings analysed to determine pre- and post-challenge means (Figure 1) and coefficients of variation (Figure 2) were 147 each for pulse rate, heart rate variability and respiratory rate. Pre- and post- challenge means (Figure 1) and coefficients of variation (Figure 2) were compared using paired parametric *t*-tests with significance set to *P* < 0.05. Correlation analysis of clinical score and the number of abnormal readings (Figure 4) were conducted using Pearson’s correlation analysis, with significance set to *P* < 0.05 and the following correlation strength definitions: r = 0.20–0.399 (weak); r = 0.40–0.599 (moderate); r = 0.60–0.799 (strong); and r = 0.80–1.000 (very strong). Comparisons of daily pulse rate, heart rate variability and respiratory rate means (Supplementary Figure 1), standard deviation (Supplementary Figure 2) and percentage of abnormal readings (Supplementary Figure 3) to the pre-challenge average were made using one-way ANOVA with Dunnett’s multiple comparisons, with significance set to *P* < 0.05.

## Results

Changes in mean pulse rate and heart rate variability (HRV) were detected by collar monitors in pigs challenged with African swine fever virus (ASFV), which were not detected by single point-in-time measurements (Figure 1). Mean pulse rate dropped significantly after challenge with ASFV compared to the pre-challenge period, as detected by multiple daily readings (*P* = 0.021; 9 out of 10 pigs); however, this reduction was not detected by daily PIT readings (*P* = 0.936; 6 out of 10 pigs) (Figure 1[a]). Similarly, mean HRV dropped significantly after challenge with ASFV compared to the pre-challenge period, as detected by multiple daily readings (*P* = 0.045; 9 out of 10 pigs); however, this reduction was also not detected by daily PIT readings (*P* = 0.178; 8 out of 10 pigs) (Figure 1[b]). Mean respiratory rate was not significantly lower post-challenge compared to the pre-challenge period, calculated from either multiple daily readings (*P* = 0.213; 6 out of 10 pigs) or single PIT readings (*P* = 0.387; 6 out of 10 pigs) (Figure 1[c]).

However, when the means of multiple daily readings were calculated and compared to daily ASF disease onset and progression, no significant daily change from baseline was observed on any day for mean pulse rate (Supplementary Figure 1[a]), HRV (Supplementary Figure 1[b]) or respiratory rate (Supplementary Figure 1[c]). Therefore, while reductions in mean pulse rate and HRV occurred post-challenge as detected by multiple daily readings, these changes were not predictive or correlative with ASF clinical disease onset or progression.

Changes in the coefficient of variation (CoV) of pulse rate and HRV were also detected by collar monitors in pigs challenged with African swine fever virus, which again were not detected by single PIT measurement (Figure 2). The variability of pulse rate increased significantly after challenge with ASFV compared to the pre-challenge period, as detected by multiple daily readings (*P* = 0.016; 8 out of 10 pigs); however, this increase was not detected by daily PIT readings (*P* = 0.264; 6 out of 10 pigs) (Figure 2[a]). Similarly, the variability of HRV readings increased significantly after challenge with ASFV compared to the pre-challenge period, as detected by multiple daily readings (*P* = 0.003; 9 out of 10 pigs); however, this increased variability was also not detected by daily PIT readings (*P* = 0.375; 8 out of 10 pigs) (Figure 2[b]). Likewise, the variability of respiratory rate increased significantly after challenge with ASFV compared to the pre-challenge period, as detected by multiple daily readings (*P* = 0.001; 9 out of 10 pigs); however, this increased variability was again not detected by daily PIT readings (*P* = 0.227; 3 out of 10 pigs) (Figure 2[c]). When the variability of multiple daily readings was calculated and compared to daily ASF disease onset and progression, a significant increase from baseline variability was observed on day 5 post-challenge for pulse rate (*P* = 0.018) (Supplementary Figure 2[a]) and HRV (*P* = 0.013) (Supplementary Figure 2[b]). Additionally, a significant increase in the daily variability of respiratory rate was observed on day 4 post-challenge (*P* = 0.026) (Supplementary Figure 2[c]). Therefore, increased variability of daily pulse rate, HRV and respiratory rate data post-challenge, as detected by multiple daily readings, correlated with ASF clinical disease onset. However, this increased data variability occurred on the same day that early clinical signs commenced and was therefore not predictive of clinical disease onset (Supplementary Figure 2[c]).

The incidence of abnormal pulse rate, HRV and respiratory rate readings increased in pigs after infection with African swine fever (Figure 3). In the pre-challenge period, 3.94% of pulse rate readings were outside of normal range, compared to 23.76% of readings post-challenge (Figure 3[a]). Of the abnormal pulse rate readings, 2.68% were higher than normal in the pre-challenge period compared to 7.89% of readings post-challenge, and 1.26% of readings were lower than normal in the pre-challenge period compared to 15.87% of readings post-challenge (Figure 3[a]). The proportion of abnormal HRV readings also increased post-challenge; 1.26% of readings were below normal in the pre-challenge period, compared to 5.6% in the post-challenge period (Figure 3[b]). Similarly, the proportion of abnormal respiratory rate readings also increased post-challenge; 0.60% of readings were below normal in the pre-challenge period, compared to 6.17% in the post-challenge period (Figure 3[c]). When the mean percentage of abnormal pulse rate readings was calculated daily and compared to daily ASF disease onset and progression, a significant increase from baseline abnormal pulse rate readings was observed on day 5 (*P* = 0.0009) and 8 (*P* = 0.037) post-challenge (Supplementary Figure 3[a]). No significant increases in mean abnormal heart rate variability readings from baseline were observed on any day (Supplementary Figure 3[b]). Significant increases in mean abnormal respiratory rate readings were observed on day 3 (*P* = 0.021) and 5 (*P* = 0.045) post-challenge (Supplementary Figure 3[c]). Therefore, increased mean abnormal readings of daily pulse rate and respiratory rate post-challenge, as detected by multiple daily readings, correlated with ASF clinical disease onset. Additionally, increased mean abnormal respiratory rate readings occurred prior to the first detection of clinical ASF disease.

Correlation analysis revealed that the number of abnormal pulse rate, HRV and respiratory rate readings increased prior to and during clinical disease onset in pigs infected with highly pathogenic African swine fever (Figure 4). A significant, very strong positive correlation was observed between clinical disease score and low respiratory rate (*P* = 0.0004; r = 0.82), and a significant, moderately positive correlation was observed between clinical disease score and both low pulse rate and low heart rate variability (*P* = 0.036; r = 0.56) (Figure 4). A significant, strong positive correlation was observed between clinical disease score and total combined daily abnormal readings (*P* = 0.034; r = 0.64) (Figure 4). In addition to these positive correlations, the number of abnormal readings appeared to increase after challenge but prior to the onset of clinical disease. This was observed in both the total number of combined abnormalities per day in relation to mean pig clinical score (Figure 4), and when comparing the clinical disease progression and abnormal readings from individual pigs for pulse rate (Supplementary Figure 4), heart rate variability (Supplementary Figure 5) and respiratory rate (Supplementary Figure 6). Additionally, trends and combinations of the type of abnormal clinical readings varied amongst pigs.

**Figure 5.**
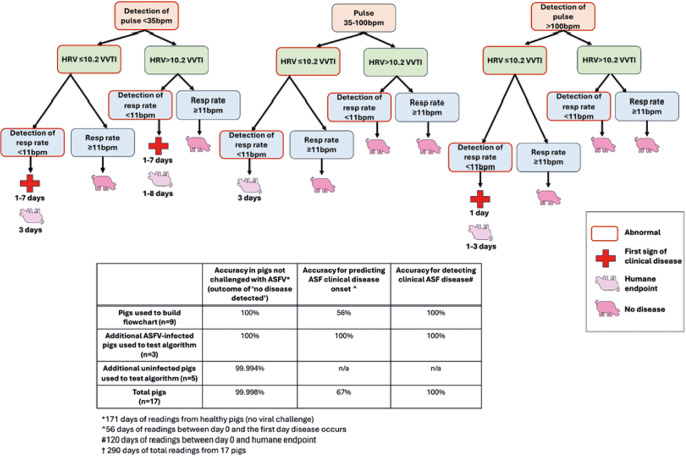
Pulse rate, heart rate variability (measured as vasovagal tonus index) and respiratory rate measurements taken from pigs wearing modified PetPace^TM^ collar monitors between 0800–1600h. Uninfected pigs (n = 5) and pigs prior to ASF challenge (n = 12) were utilised to test the algorithm in healthy pigs, with one pig on one day incorrectly predicted to develop African swine fever (ASF) clinical disease (from total n = 171 days of readings). N = 9 pigs were confirmed to have become infected via primary inoculation of ASF virus and were used to develop the algorithm. An additional three pigs that became infected and developed ASF clinical disease were subsequently utilised to test the algorithm (n = 2 that became infected via secondary contact transmission, n = 1 pig that was humanely killed prior to reaching humane endpoint). All twelve pigs had ASF disease detected after challenge via the algorithm prior to (n = 8) and during (n = 12) clinical disease presentation.

Based on these analyses of pulse rate, HRV and respiratory rate throughout the course of infection, a preliminary, non-AI algorithm was developed which predicted and detected highly pathogenic ASF disease in pigs (Figure 5). When combined abnormalities of pulse rate, HRV and/or respiratory rate were applied via the algorithm to pigs challenged with ASFV, ASF disease was detected in 100% of pigs between challenge and humane endpoint (12 out of 12 pigs) (Figure 5). Additionally, of these 12 pigs, 67% (8 out of 12) were detected as being infected with ASF prior to the onset of clinical disease (Figure 5). Out of a total of 171 days of readings from healthy pigs that had not been challenged with ASFV, 170 days were correctly detected as ‘no ASF disease’ (an accuracy of 99.998%). A pseudocode flowchart to facilitate written code for an automated algorithm, and to allow for further refinement and application of this preliminary algorithm, is presented in Supplementary Figure 7.

## Discussion

The pulse rate, heart rate variability (HRV) and respiratory rate of pigs were measured throughout two studies of ASF infections in pigs, using health-monitoring collars for regular, automatic monitoring. Data were then analysed to investigate if changes in these variables throughout the course of infection detected and predicted ASF disease. When assessing multiple daily data recordings, collar monitors (n = 10 pigs) detected significantly reduced means (pulse rate and HRV) and significantly increased variability in readings (pulse rate, HRV and respiratory rate) after viral challenge, compared to data collected prior to viral challenge. In contrast, the means and variability of pulse rate, HRV and respiratory rate were not significantly different pre- and post-challenge when only a daily point-in-time (PIT) reading was assessed. The present study is the first to demonstrate the cardiorespiratory responses of pigs over the course of ASF infection and demonstrates the value of using health-monitoring devices to increase data collection and detect early physiological changes. While this is also the first study to demonstrate the HRV of pigs infected with ASF, the mean reduction (and increased variability as a result of a greater number of lower HRV readings) is not unexpected, as reduced heart-rate variability is a known response to physiological stressors in animals (Jarkovska *et al.* 2017; Halachmi *et al.* 2019). Previous studies and observations of ASF-infected pigs have reported increased pulse and respiratory rates in the final stages of disease (within two days of death), which is in contrast to the reduced mean pulse and respiratory rates observed post-challenge in the present study (Prutsakov *et al.* 2015; Hess 2019). This may be due to pigs in the present study being humanely killed at an endpoint prior to the development of consistently high pulse and respiratory rates. While no previous studies have conducted regular monitoring of these parameters in pigs during ASF infection, extrapolation of heart and respiratory rate data in other haemorrhagic animal disease models provides further insight into the findings of the present study. Ebola is another severe haemorrhagic viral disease where increased pulse and respiratory rates are reported during late-stage disease (Poliquin *et al.* 2019; Blair *et al.* 2021). To investigate the cardiorespiratory responses of rhesus macaques (*Macaca mulatta*) to infection with Zaire ebolavirus, Kortepeter *et al.* (2011) implanted animals (n = 9) with telemetry real-time monitoring devices, followed by viral challenge 30 days later. While the authors found that pulse and respiratory rates increased at peak infection (and remained high in macaques that reached humane endpoint), telemetry data from this study also detected reduced pulse and respiratory rates from baseline in early to mid-stage infection (Kortepeter *et al.* 2011). This is consistent with findings in the present study, where reduced means and increased variation in pulse and respiratory rates post-ASF challenge were observed. This was due to changes in two-way trends (not just increases) in these variables over the course of infection, potentially as a result of viral-mediated autonomic dysfunction which has been observed in other viral diseases (Carod-Artal 2018). As abnormal readings are sporadic and occur amongst normal readings, these changes can only be reliably detected via ongoing, preferably undisturbed, monitoring. This ensures that intermittent abnormal readings and changes from baseline are detected, as abnormal readings may not be present at the time of single PIT measurements.

In addition to assessing the means and variability of pulse rate, HRV and respiratory rate, the percentage of abnormal readings pre- and post-challenge were also investigated. The incidence of abnormal readings increased after viral challenge for pulse rate (both abnormally high and abnormally low readings), HRV (abnormally low readings) and respiratory rate (abnormally low readings). These increases in abnormal readings are a key factor in the reduced means and increased data variability observed in pigs post-challenge and occur as a result of physiological disruptions caused by disease and inflammation (Liu *et al.* 2019; Mejía-Mejía *et al.* 2020). While pulse, HRV and respiratory measures have not previously been investigated for the detection of ASF in pigs, a recent study by Morelle *et al.* (2023) assessed the use of accelerometers for regular, automatic activity logging to detect ASF in boars. Accelerometer data were collected both pre- and post-challenge with ASFV. The authors found that activity levels in boars reduced by 10–20% in the ASF viraemia phase, suggesting that accelerometers may provide an early indication of ASFV infection prior to the onset of clinical signs and viral shedding (Morelle *et al.* 2023). While the metrics under investigation are different, the results from this study by Morelle *et al.* (2023) align with those of the present study. When the incidence of cardiorespiratory abnormalities is assessed across study days and compared against mean clinical disease score, the incidence of abnormal readings correlates with clinical disease progression, and in some instances, increases prior to clinical disease onset. For example, changes in HRV have been previously reported to occur prior to other indicators of disease, as described in a sepsis study by Jarkovska *et al.* (2016. The authors measured the HRV of pigs (n = 11) before and after the induction of moderate sepsis via faecal peritonitis. They observed a reduction in HRV prior to the onset of sepsis-associated haemodynamic changes, suggesting that HRV reduction may serve as an early indicator of sepsis-related systemic inflammation (Jarkovska *et al.* 2016. While a decreased HRV was also observed in some pigs in the present study, the presence of abnormally low HRV readings alone was not sufficient for reliably predicting or detecting ASF clinical disease. This is due to the relatively low number of abnormal readings detected, and the presence of abnormal readings prior to viral challenge on the day of arrival (likely due to stress, which is known to reduce HRV) (Von Borell *et al.* 2007; Brandt & Aaslyng 2015. An increased incidence of abnormal pulse and respiratory rate readings also occurred after challenge with ASFV in the present study, but abnormalities in each metric alone did not occur exclusively post-challenge. This presents an opportunity to assess the observed cardiorespiratory abnormalities in combination, for a more robust detection and prediction of ASF disease.

Combined abnormalities in pulse rate, HRV and respiratory rate pre- and post-infection were assessed, in order to develop a non-artificial intelligence (non-AI) algorithm for the detection and prediction of ASF disease. The algorithm correctly identified healthy pigs (n = 171 days of healthy pig data) with 99.998% accuracy, detected pigs (n = 12) presenting with clinical signs of ASF disease with 100% accuracy, and predicted impending clinical disease onset in pigs (n = 12) with 67% accuracy. The accuracy of this non-AI algorithm is consistent with algorithm accuracy using AI-based methods in the literature, as described in a study by Ellmann *et al.* (2020). The authors combined magnetic resonance imaging (MRI) features of tissues vascularisation and positron emission tomography/computed tomography (PET/CT) of glucose metabolism in rats (*Rattus norvegicus*), with and without induced macrometastatic bone disease. When this combination of data was used to develop a machine-learning algorithm, macrometastatic bone disease development was predicted with an accuracy of 60–80% (Ellmann *et al.* 2020). Other machine learning algorithms of disease onset and prediction have been shown to have an accuracy of between 90–100%, demonstrating the potential effectiveness of AI algorithms for animal monitoring (Mei *et al.* 2019; Carslake *et al.* 2020). However, the large data-sets required to develop and train AI algorithms (particularly using machine learning) is often prohibitive and requires specific training and expertise (Sarker 2021). The algorithm developed in the present study demonstrates how a moderate amount of data can be utilised to develop a non-AI algorithm. This can improve the detection and prediction of disease in research animal disease models, without the requirement for expertise in scientific computing and machine learning.

### Animal welfare implications

This non-AI algorithm enhances the ability of animal care staff and researchers without a scientific computing background to detect and predict ASF disease onset and progression. While not a replacement for stringent visual monitoring and clinical scoring, this algorithm provides an additional opportunity to strengthen animal monitoring for enhanced welfare outcomes. Gathering physiological data from additional ASF pig studies will lead to further refinement of this algorithm, with the potential to identify patterns to predict impending humane endpoints or progression to severe disease. Whilst the algorithm presented in this study is specific to highly pathogenic ASF, this algorithm framework and methodology also has the potential to be adapted across different diseases and species. As new monitoring technologies are developed, implantable devices capturing multiple physiological metrics may be utilised for further algorithm development, refinement, and use. This could provide an opportunity to continually monitor group-housed research animals without the risk of device damage, reducing or eliminating the need for staff supervision and allowing data to be collected up to 24 h a day. Smaller devices that could be implanted using an applicator, rather than via a surgical procedure, would provide additional future potential to utilise this algorithm framework for animals outside of the laboratory, such as on-farm sentinels. This could enhance the detection of ASF and other infectious diseases at an early stage for enhanced biosecurity and animal welfare outcomes.

## Supporting information

10.1017/awf.2026.10060.sm001 Layton et al. supplementary material Layton et al. supplementary material

## Supplementary material

The supplementary material for this article can be found at http://doi.org/10.1017/awf.2026.10060.
