# Supplementary material for: A non-AI preliminary algorithm for the prediction and detection of highly pathogenic African swine fever in pigs using health monitoring collars
Source: Anim Welf. 2026 Jan 28;35:e8. doi: 10.1017/awf.2026.10060 (PMC12895198; doi:10.1017/awf.2026.10060)
Supplement: Layton et al. supplementary material [file S0962728626100608sup001.zip › Supplementary Figure 1.pdf]

a) **Pulse rate**

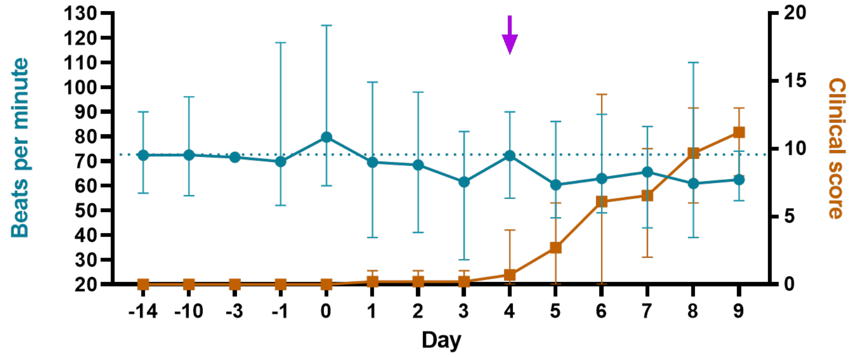

b) **Heart rate variability**

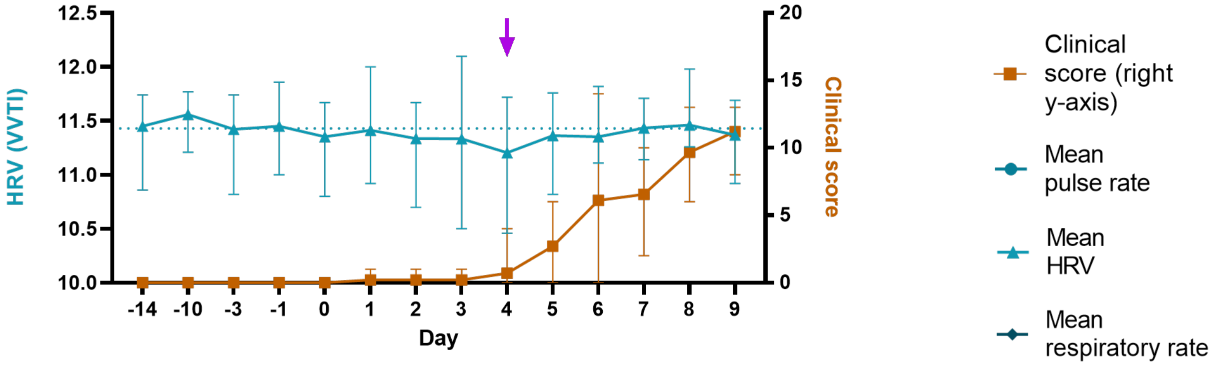

c) **Respiratory rate**

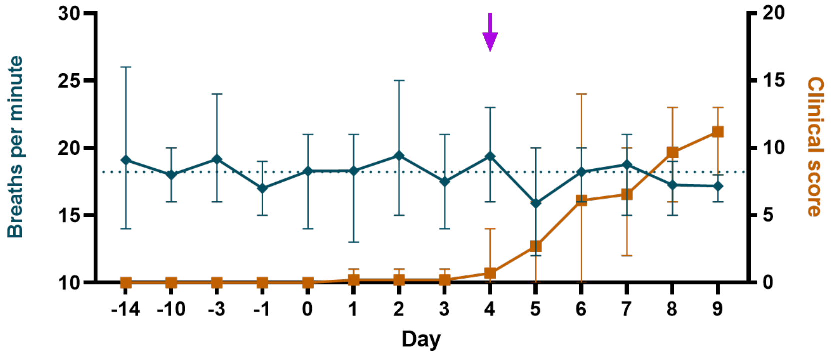

Supplementary figure 1: Mean daily pulse rate, heart rate variability and respiratory rate is not predictive or correlative with clinical disease onset, viral shedding or viraemia in pigs infected with African swine fever. Means were calculated from all readings collected for each pig (n=9) per day of pulse rate (a), heart rate variability (b) and respiratory rate (c). Each day pre- and post-challenge was compared to the average pre-challenge value, represented on each graph by the dotted horizontal line. ↓ = first detection of oral shedding and viraemia. Error bars represent the range. Comparisons were made of each day to the pre-challenge average using one-way ANOVA with Dunnett's multiple comparisons, ns =p>0.05.
