# Supplementary material for: A non-AI preliminary algorithm for the prediction and detection of highly pathogenic African swine fever in pigs using health monitoring collars
Source: Anim Welf. 2026 Jan 28;35:e8. doi: 10.1017/awf.2026.10060 (PMC12895198; doi:10.1017/awf.2026.10060)
Supplement: Layton et al. supplementary material [file S0962728626100608sup001.zip › Supplementary Figure 3.pdf]

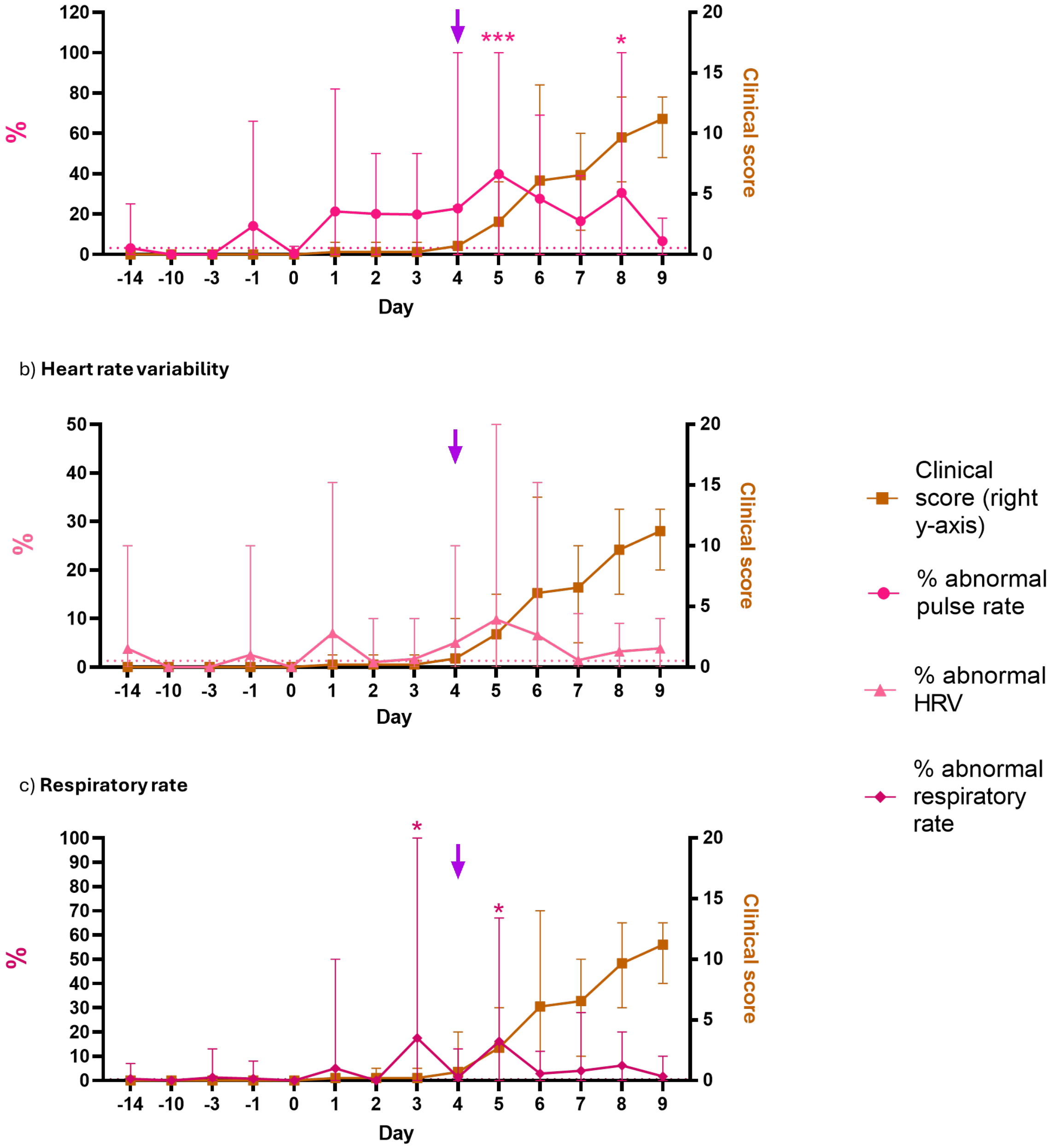

Supplementary figure 3: Increased abnormal respiratory rate occurs prior to the onset of clinical disease, oral shedding and viraemia in pigs infected with African swine fever. Percentage of abnormal readings was calculated from all readings collected for each pig (n=9) per day of pulse rate (a), heart rate variability (b) and respiratory rate (c). Each day pre- and post-challenge was compared to the average pre-challenge value, represented on each graph by the dotted horizontal line. ↓ = first detection of oral shedding and viraemia. Error bars represent the standard error of the mean. Comparisons were made of each day to the pre-challenge average using one-way ANOVA with Dunnett's multiple comparisons, *= p<0.05, **p<0.01, ***p<0.001.
