# Supplementary material for: A non-AI preliminary algorithm for the prediction and detection of highly pathogenic African swine fever in pigs using health monitoring collars
Source: Anim Welf. 2026 Jan 28;35:e8. doi: 10.1017/awf.2026.10060 (PMC12895198; doi:10.1017/awf.2026.10060)
Supplement: Layton et al. supplementary material [file S0962728626100608sup001.zip › Supplementary Figure 5.pdf]

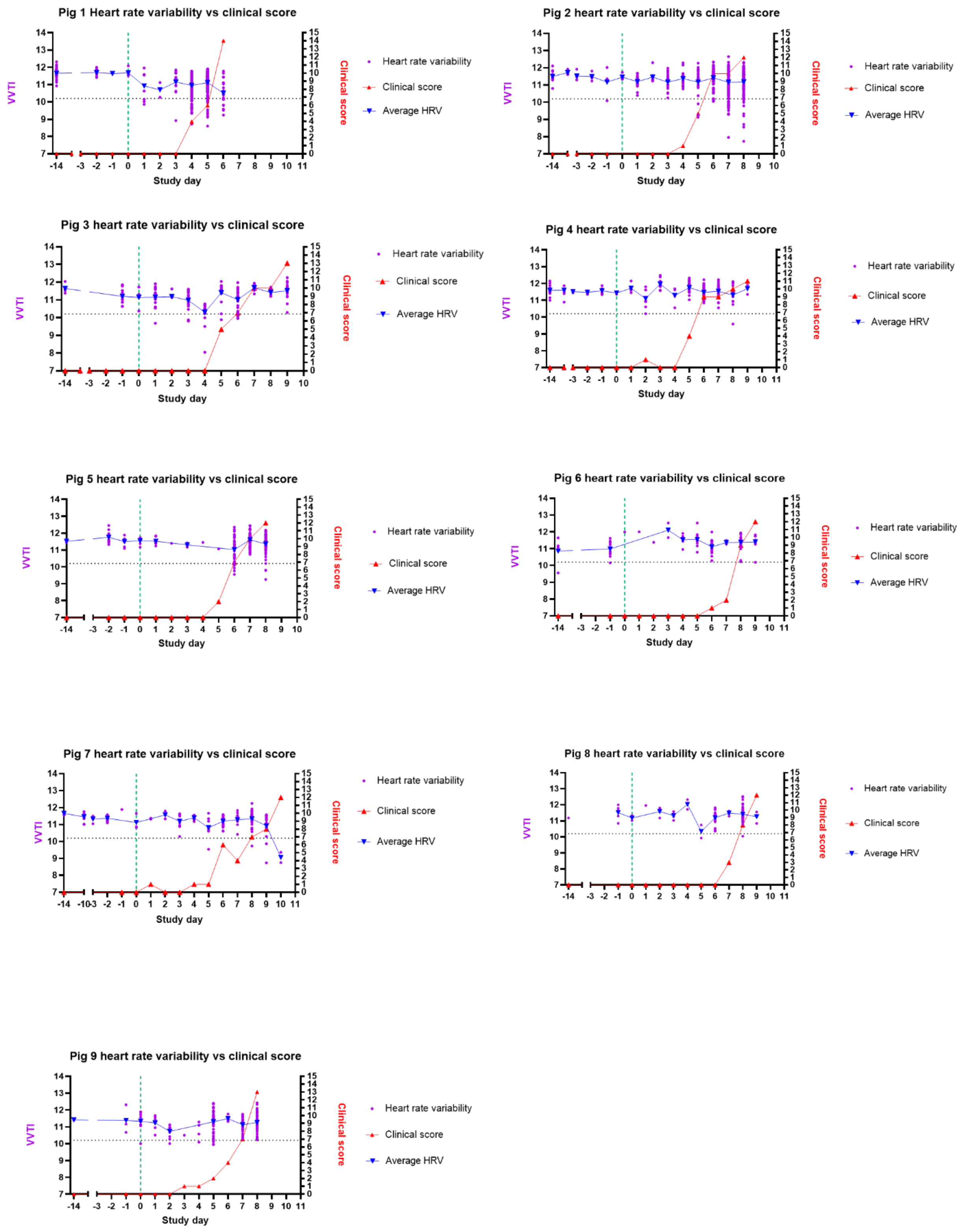

Supplementary figure 5: Individual pig heart rate variability and clinical disease scores throughout African swine fever disease studies. All heart rate variability values collected as VVTI via modified PetPace collar monitors. Pigs arrived day -14. Green dotted vertical line represents day of viral challenge. Black dotted horizontal line represent the lower normal heart rate variability for growing healthy pigs housed under similar laboratory conditions.
