# Supplementary material for: A non-AI preliminary algorithm for the prediction and detection of highly pathogenic African swine fever in pigs using health monitoring collars
Source: Anim Welf. 2026 Jan 28;35:e8. doi: 10.1017/awf.2026.10060 (PMC12895198; doi:10.1017/awf.2026.10060)
Supplement: Layton et al. supplementary material [file S0962728626100608sup001.zip › Supplementary Figure 6.pdf]

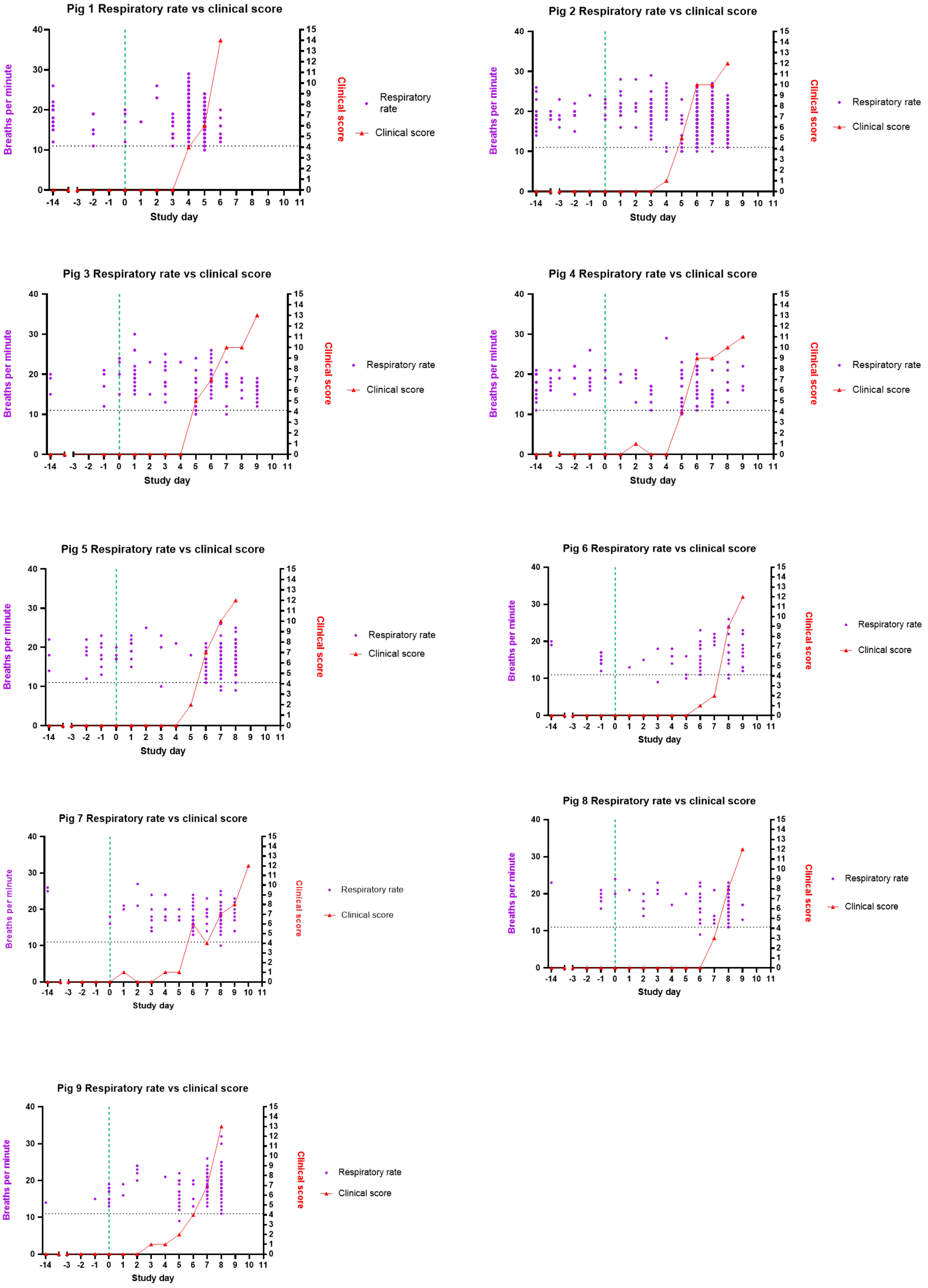

Supplementary figure 6: Individual pig respiratory rate and clinical disease scores throughout African swine fever disease studies. All respiratory rate values collected via modified PetPace collar monitors. Pigs arrived day -14. Green dotted vertical line represents day of viral challenge. Black dotted horizontal line represent the lower normal respiratory rate for growing healthy pigs housed under similar laboratory conditions.
